# Editorial: Innovative personalized medicine strategies for managing multi-morbidity

**DOI:** 10.3389/fmed.2026.1947718

**Published:** 2026-09-17

**Authors:** Priyank Shukla, Catriona Kelly, Victoria McGilligan, David S. Gibson

**Affiliations:** Personalised Medicine Centre, School of Medicine, Ulster University, C-TRIC Building, Altnagelvin Area Hospital, Londonderry, United Kingdom

**Keywords:** comorbidity, MLTC, multimorbidity, multiple long-term conditions, personalized medicine

Multimorbidity is defined as the co-occurrence of two or more multiple long-term conditions (MLTC) and is associated with increased use of health services and reduced quality of life and life expectancy (1). It is becoming more prevalent, especially among individuals over the age of 65 where two-third of the world population is affected (1). Chronic pain, reduced mobility, limited social opportunities, and mental health challenges are some common issues reported by those living with MLTC (2). This research topic aimed to collate original research, expert views and novel personalised medicine approaches and strategies focused on improving multi-morbidity management. It includes eleven articles from key opinion leaders, ranging from three reviews, one systematic review, one brief research report and six original research articles.

In the context of the topic theme, two of the four review articles were primarily focused on computational methods and their application in multimorbidity studies. More specifically, review article by Woodman et al., provided an overview of the application of graph theory for studying multimorbidity. They reviewed the use of knowledge graphs, graph algorithms, graph embedding methods, and graph machine learning within the context of multimorbidity and the construction of disease comorbidity networks. Importantly, they present examples of the application of knowledge graphs for patient diagnosis, adverse drug reaction prediction, and treatment recommendation. The other article by Chen et al. focused on systematically reviewing risk prediction models for mortality in multimorbid patients and presented a meta-analysis by deploying Prediction Model Risk of Bias Assessment Tool (PROBAST) to evaluate the risk of bias and clinical applicability of the published models. Age and body mass index (BMI) were found to be the most common predictors across the studies. While all models showed discriminatory ability in predicting mortality, all exhibited significant risks of bias based on the PROBAST assessment. Rigorous study designs and multi-centre external validation were recommended for improving the precision of risk predictions.

The other two review articles focused on highlighting the importance of considering multimorbidity as a key variable when developing personalised medicine treatment strategies for an index disease or health condition. Particularly, Qi et al's review on personalised peripheral vascular interventional embolization in tumour treatment suggests patient-specific factors such as comorbidities may influence personalised treatment decisions and hence should be carefully evaluated. The authors also note that while heterogeneity in comorbidity assessment makes cross-study comparison difficult, consensus exists on multimorbidity as a key factor for personalising treatment and need for standardised protocols. The other review article from Volaric et al. highlights the role of chronic stress in the development of multimorbidity through increasing allostatic load. Their article synthesises the information on allostatic load differences connected to sex/gender and age with an aim to provide a model for future studies on allostatic load, with a focus on these differences.

Patient stratification at the primary care level has been proposed as the first step to tackle MLTCs effectively (4), and multimorbidity trajectories have been analysed to understand sex-specific impacts on mortality and re-hospitalisation risk (5). In a continuation of these approaches, Liu et al. investigated five waves of national surveys from the China Health and Retirement Longitudinal Study (CHARLS) to identify distinct multimorbidity patterns and model the trajectory and dynamic evolution of multimorbidity over time. Their findings from this longitudinal study data suggest disease burden among older adults in China has shifted from gastrointestinal metabolism disorders to cardiovascular diseases, with the former acting as precursors to the latter. Yin et al. investigated the same dataset to reveal an association between decreased sleep duration and unfavourable sleep trajectories with higher odds of overall multimorbidity in Chinese middle-aged and older adults. This association was particularly pronounced in adults with respiratory and arthritis diagnoses, alongside digestive and multi-system conditions.

Assessment of quality of life of multimorbid patients is a key data informing healthcare policies. In this regard, Sahoo et al. presented a brief research report article wherein they assessed the health-related quality-of-life (HRQoL) data from an observational study of multimorbid patients recruited at tertiary healthcare facilities in an Indian state. Their findings suggest a correlation between HRQoL and multimorbidity in older adults, highlighting implications for healthcare systems and clinical and policy decisions. Another cross-sectional study from Su et al. was conducted on community-dwelling older adults from two randomly selected cities in China. The Pittsburgh Sleep Quality Index (PSQI) was used to assess sleep quality, which was found to be poor in older adults, with women reporting poorer quality than men, and multimorbidity cited as the biggest challenge causing difficulty in falling asleep.

As stated earlier, multimorbidity is primarily associated with the elderly. Two of the contributions focused on geriatric assessment tools and scores. In particular, Cho et al. examined the presence of multimorbidity and specific geriatric syndromes in older adults across one of the states of US with the Rapid Geriatric Assessment (RGA) tool. This cross-sectional study revealed individuals with sarcopenia were over three times more likely to have multimorbidity compared to those without sarcopenia, evidencing RGA as a valid screening tool for multimorbidity assessment. The exploratory study by Wu et al. investigated intestinal microbiota, clinical variables, and 18-month mortality in long-living male patients undergoing multiple medications. Their results suggest Cumulative Illness Rating Scale for Geriatrics (CIRS-G) scores and serum creatinine levels have prognostic potential for predicting mortality among long-living male patients. However, they acknowledge that small sample size, monocentric design, and potential confounders limit generalisability of their findings.

Current treatment strategies focus predominantly on individual diseases often resulting in prescribing multiple medications, with increased contraindication risk, medication side effects and adverse outcomes. Personalised medicine, which aims to customise treatments based on an individual's characteristics, is challenged by the complexity of medication prescribing in people living with MLTC. In this regard, a study on multimorbidity and polypharmacy was conducted by Kuri et al. who analysed the level of polypharmacy i.e., multiple comedications, in an isolated rural population on the island of Tenerife, Spain. The authors found age and initial polypharmacy level were key cofactors, along with multimorbidity in some cases, which influences final polypharmacy levels amongst patients. The study, although limited by a modest sample size of seventy-eight participants, provides valuable insight into the growing problem of polypharmacy which is widely associated with multimorbidity.

In 2018, the Academy of Medical Sciences identified multiple long-term conditions as a priority for global health research (3). The contributions to this Research Topic demonstrate both the complexity of multimorbidity and the potential for personalised medicine to move its management beyond the traditional single-disease approach. However, heterogeneity in how multimorbidity and comorbidities are defined, assessed and reported remains a major barrier to comparing findings across studies and populations. Developing standardised approaches to multimorbidity assessment and reporting should therefore be a priority to guide future research, enable cross-study comparisons and support translation into clinical practice. Longitudinal studies across diverse populations, alongside robust external validation of emerging computational and risk-prediction approaches, will also be important.

A particularly promising opportunity lies in addressing polypharmacy through more personalised prescribing and deprescribing strategies, potentially incorporating pharmacogenomics, biomarkers and clinical decision-support tools. Ultimately, multimorbidity research must progress from describing disease patterns and associations towards developing and testing interventions that improve outcomes that matter to patients, including quality of life, treatment burden and functional independence. Such approaches could help shift multimorbidity care from the reactive management of individual conditions towards proactive, integrated and genuinely personalised healthcare.
